# The complete chloroplast genome of Eastern Asian fern *Bolbitis laxireticulata* (Dryopteridaceae)

**DOI:** 10.1080/23802359.2023.2171693

**Published:** 2023-02-10

**Authors:** Yu-Jie Liao, Li-Yun Nie, Xin-xin Cheng, Ai-Hua Wang, Lei Duan, Wen-Chao Zhong, Fa-Guo Wang

**Affiliations:** a Key Laboratory of Plant Resources Conservation and Sustainable Utilization, South China Botanical Garden, Chinese Academy of Sciences, Guangzhou, China; b College of Life Sciences, University of Chinese Academy of Sciences, Beijing, China; c Key Laboratory of Environment Change and Resources Use in Beibu Gulf, Ministry of Education, Nanning Normal University, Nanning, China; d Nankunshan Provincial Nature Reserve Management of Longmen, Huizhou, China

**Keywords:** *Bolbitis laxireticulata*, chloroplast genome, Dryopteridaceae

## Abstract

*Bolbitis laxireticulata* is a potential ornamental plant, which is restricted to eastern Asia. Here, we sequenced the complete chloroplast (cp) genome of *B. laxireticulata* and constructed a phylogenetic cp tree of Dryopteridaceae to study their relationships. The cp genome of *B. laxireticulata* is 153,093 bp in length, being made up of large single-copy (LSC, 83,169 bp), small single-copy (SSC, 21,538 bp), and a pair of region inverted repeats (IRs, 24,193 bp). It has 124 genes including 83 protein-coding genes, 33 tRNA genes, and eight rRNA genes. With the maximum-likelihood tree indicating, *B. laxireticulata* is more closely related to *B. subcordata*.

The genus *Bolbitis* Schott (Dryopteridaceae) is distributed in tropical and subtropical regions which includes around 80 species (Schuettpelz 2016). With viviparous apices of fronds and variable shape that resembles both *Bolbitis sinensis_* (Baker) K. Iwatsuki 1959 and *B. singaporensis_*Holttum 1928, *B. laxireticulata_* K. Iwatsuki 1959 has higher ornamental value, and restricted to Guangdong, Hainan, Taiwan (China) and Ryukyu Islands (Japan) (Iwatsuki 1959; Dong and Zhang 2005). It is the first time to sequence the cpDNA of *B. laxireticulata.* Here, we constructed a chloroplast (cp) maximum-likelihood (ML) tree and aimed to explore its genetic relationships with other species in *Bolbitis*. Research on the relationship of those species can not only provide necessary evidence for combing some complex system evolutionary relationships, but also can solve hybrid transition form and the classification of the difficult problems that cause. This study provides essential basic data for the formation and reticulate evolution of the *Bolbitis*, and provides theoretical basis and technical support for the further work of species conservation and breeding.

The fresh leaf materials of *B. laxireticulata* were collected in Longmen County (longitude: 113°28′52″E, latitude: 23°54′09″N, altitude: 550 m), Guangdong Province, China (Figure 1(A,B)). The voucher specimen was deposited at Herbarium of South China Botanical Garden, Chinese Academy of Sciences (IBSC) (http://herbarium.scbg.cas.cn/, Feiyan Zeng, zengfeiy@scbg.ac.cn), under the voucher number: WFG6462. CTAB methods were used to extract the total genomic DNA from the leaf tissue samples (Doyle 1987). Preparation and sequencing of genomic libraries used the Illumina NovaSeq 6000 platform (Illumina Inc., San Diego, CA) with 150 bp end lengths. The resultant sequences were filtered and the raw data were removed by Trimmomatic (Bolger 2014). The adaptor-free reads were then assembled with SPAdes v3.11 (Bankevich et al. 2012). GeSeq v2.03 (Michael 2017) was used to annotate the cp genome. Then, the genome was deposited in GenBank (accession number: OM350395).

**Figure 1.**
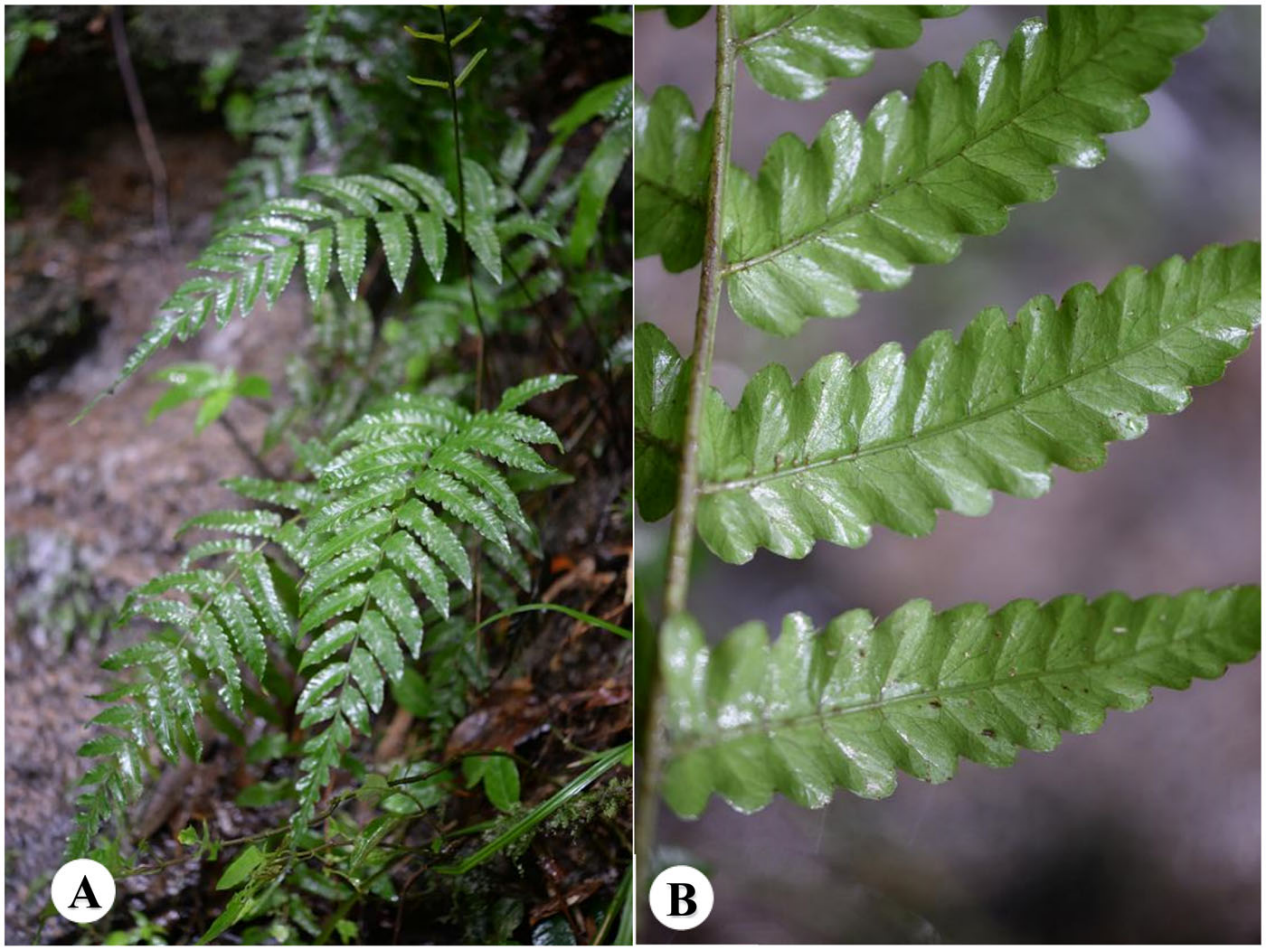
Species reference images of *Bolbitis laxireticulata.* (A) Plant shape of *B. laxireticulata*. (B) Morphological characteristics of leaves of *B. laxireticulata* (lobes varied in length and irregular, reticulate veins conspicuous). The species photo was taken by the author in Nankunshan Nature Reserve, Longmen Country, Huizhou, China, August 2021, without any copyright issues.

The raw reads of *B. laxireticulata* are approximately 6 G, and its cp genome is 153,093 bp in length, including a large single-copy (LSC, 83,169 bp), a small single-copy (SSC, 21,538 bp), and a pair of inverted repeats (IRs, 24,193 bp). Totally, 124 genes were annotated, including 83 protein-coding genes, 33 tRNA genes, and eight rRNA genes, within which 14 genes (*atpF*, *ndhA*, *petB*, *petD*, *rpl2*, *rpl1*6, *rpoC1*, *rps12*, *rps16*, *trnA*-*UGC*, *trnG*-*UCC*, *trnI*-*GAU*, *trnL*-*UAA*, *trnA*-*UAC*) had one intron, two genes (*clpP*, *ycf3*) had two introns (Figure 2). The overall G/C content of total genome is 42.3%.

**Figure 2.**
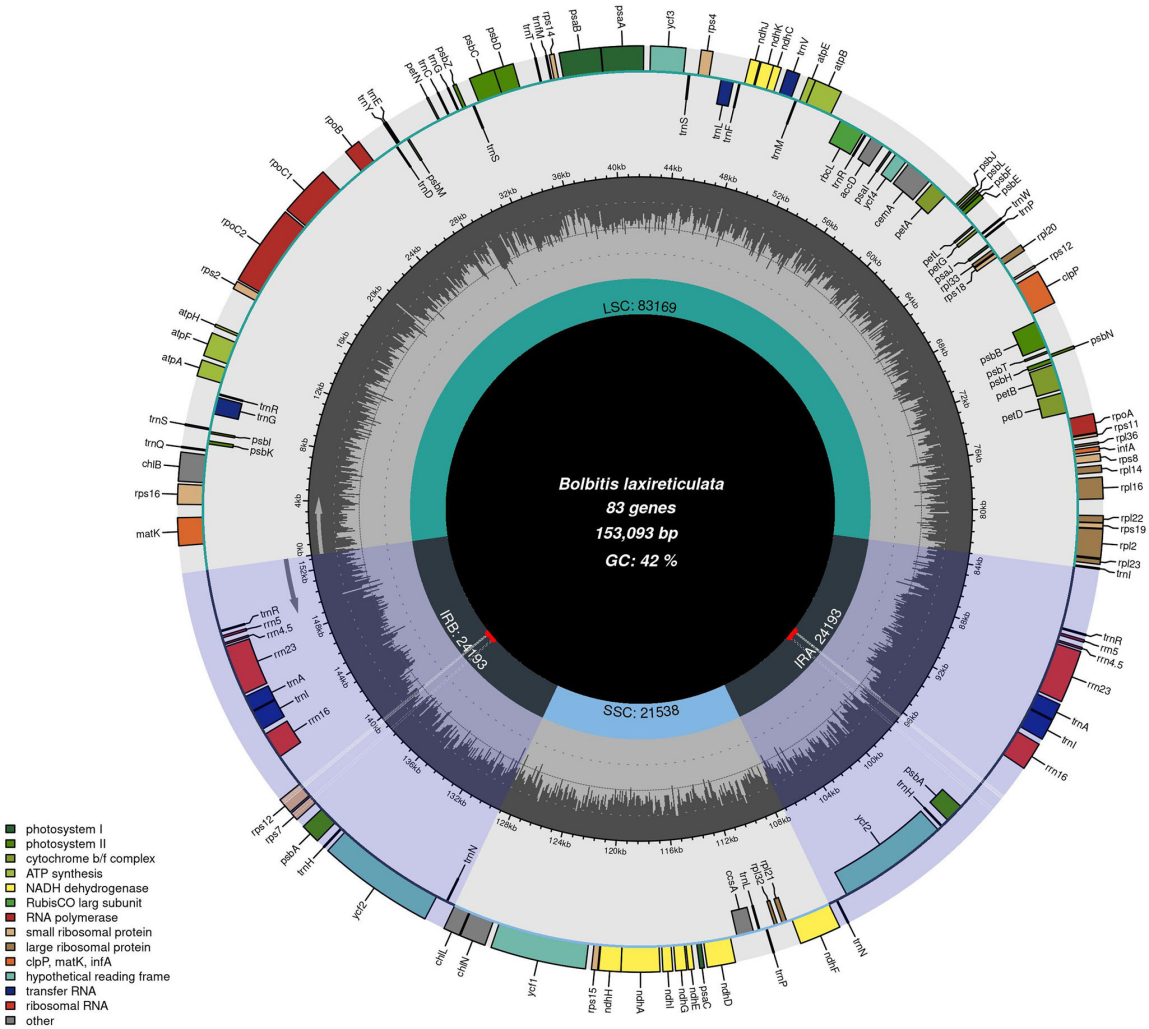
The chloroplast genome map of *B. laxireticulata* (OM350395).

In order to understand the phylogenetic relationship of *B. laxireticulata*, we downloaded 11 cp genomes from GenBank to build phylogenetic relationship in a phylogram (Figure 3). *Pteris multifida_* Poir 1804 was chosen as the outgroup. Geneious prime and Mauve plugin were used to adjust gene order and remove a copy of the IR regions of the cp genome to detect gene rearrangements with homozygous genes. We used MAFFT v.7 (Katoh and Standley 2013) to align the 12 cp genes which is used to construct the phylogenetic tree. Then we used TrimAl (Capella-Gutierrez et al. 2009) to trim the alignment with automatic parameter. Finally, IQ-TREE v.1.4.2 (Nguyen et al. 2015) program was used to run an ML tree with 5000 bootstrap replicates (Figure 3). All GenBank accession numbers including the new obtained accession number of *B. laxireticulata* are given in Figure 3. We then generated a physical map of the cp genome using Chloroplast Genome Viewer (CPGView) (http://www.1kmpg.cn/cpgview). The ML tree shows that *B. laxireticulata* is more closely related to *B. subcordata_* (Cop.) Ching 1934 (Figure 3), and also has closer phylogenetic relationship to *B. appendiculata_* (Willdenow) K. Iwatsuki 1959 and *B. heteroclita* (Presl) Ching 1934. The next step will be to further verify the hybridization relationship of *B. laxireticulata* at macro- and micro-levels.

**Figure 3.**
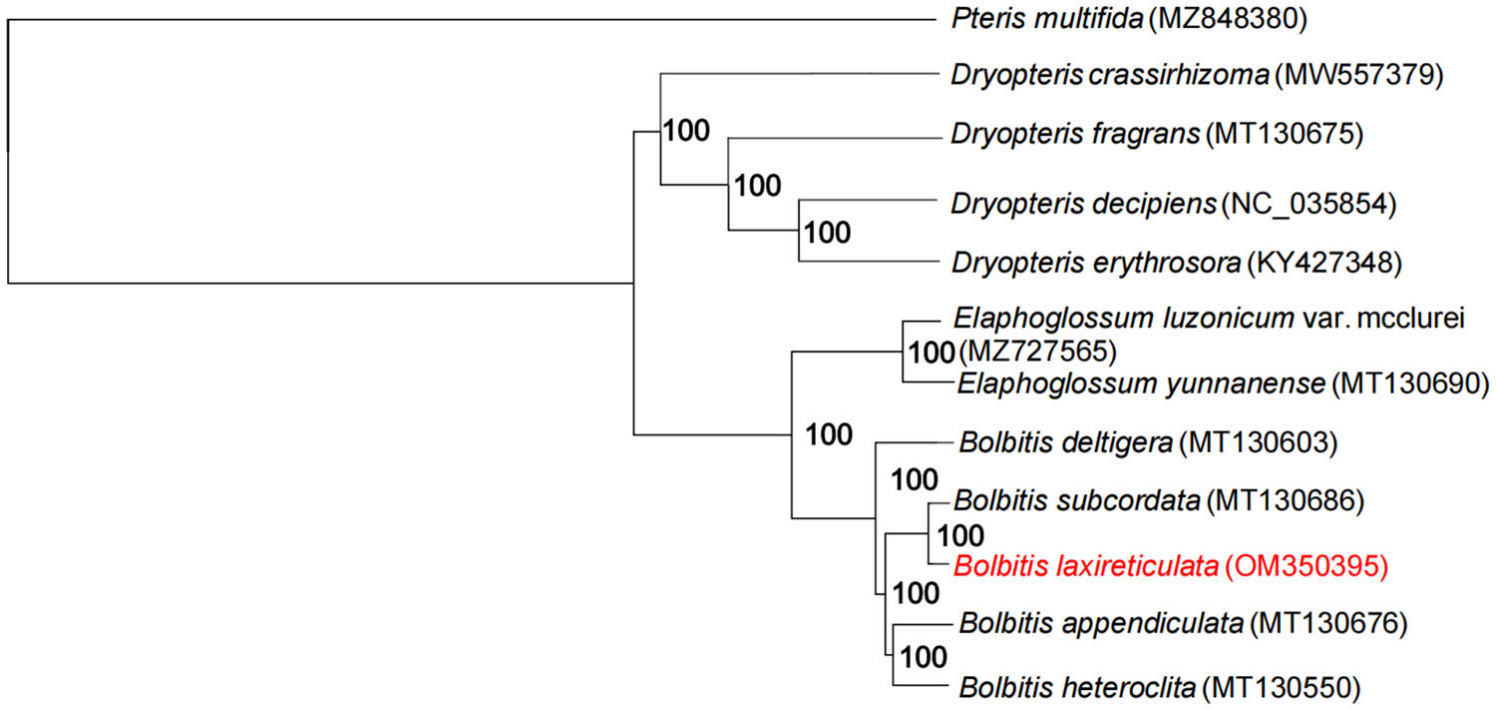
Maximum-likelihood phylogeny of *B. laxireticulata* and related taxa based on 12 complete chloroplast genomes. The maximum-likelihood bootstrap support values are along the branches.

## Data Availability

The genome sequence data that support the findings of this study are openly available in GenBank of NCBI at https://www.ncbi.nlm.nih.gov/ under the accession no. OM350395. The associated BioProject, SRA, and Bio-Sample numbers are PRJNA817290, SRR18361020, and SAMN26754306, respectively.
